# Prediction of Gestational Diabetes Mellitus: A Nomogram Model Incorporating Lifestyle, Nutrition and Health Literacy Factors

**DOI:** 10.3390/nu17213400

**Published:** 2025-10-29

**Authors:** Minghan Fu, Menglu Qiu, Zhencheng Xie, Laidi Guo, Yun Zhou, Jia Yin, Wanyi Yang, Lishan Ouyang, Ye Ding, Zhixu Wang

**Affiliations:** 1 Department of Maternal, Child and Adolescent Health, School of Public Health, Nanjing Medical University, Nanjing 211166, China; fuminghan@stu.njmu.edu.cn (M.F.); menglu-qiu@stu.njmu.edu.cn (M.Q.); zhenchengxie@njmu.edu.cn (Z.X.); guolaidi@stu.njmu.edu.cn (L.G.); zygw@stu.njmu.edu.cn (Y.Z.); yinjia0819@stu.njmu.edu.cn (J.Y.); yangwanyi@stu.njmu.edu.cn (W.Y.); ouyanglishan@stu.njmu.edu.cn (L.O.); 2 The Institute of Nutrition and Food Science, Nanjing Medical University, Nanjing 211166, China

**Keywords:** gestational diabetes mellitus, prediction model, Chinese pregnant women, lifestyle, nutrition and health literacy

## Abstract

**Background:** Over the past several decades, the prevalence of gestational diabetes mellitus (GDM) has risen markedly worldwide, posing serious threats to both maternal and child health by increasing adverse pregnancy outcomes and long-term metabolic risks. Developing effective risk prediction tools for early detection and intervention has become the most important clinical priority in this field. The current GDM prediction models primarily rely on non-modifiable factors, for example age and body mass index, while modifiable factors such as lifestyle and health literacy, although strongly associated with GDM, have not been fully utilized in risk assessment. This study sought to establish and validate a nomogram prediction model combining modifiable and non-modifiable risk factors, with the goal of identifying high-risk Chinese pregnant women with GDM at an early stage and promoting targeted prevention and personalized prenatal management. **Methods:** A multicenter study was conducted across 7 maternal health institutions in Southern China (2021–2023), enrolling 806 singleton pregnant women (14–23^+6^ weeks). The collected data included sociodemographic, clinical history, and modifiable factors collected through validated questionnaires: dietary quality, physical activity level, sleep quality, and nutrition and health literacy. GDM was diagnosed via 75 g oral glucose tolerance test at 24–28 weeks. Predictive factors were identified through multi-variable logistic regression. A nomogram model was developed (70% modeling group) and validated (30% validation group). Receiver operator characteristic curves, calibration curves, and decision curve analysis were used to evaluate the prediction ability, the degree of calibration, and the clinical benefit of the model, respectively. **Results:** The finalized risk prediction model included non-modifiable factors such as maternal age, pre-pregnancy weight, and maternal polycystic ovary syndrome, as well as modifiable factors including dietary quality, physical activity level, sleep quality, nutrition and health literacy. The application of the nomogram in the modeling group and the validation groups showed that the model had high stability, favorable predictive ability, good calibration effect and clinical practicality. **Conclusions:** Overall, the integrated model demonstrates significant clinical utility as it facilitates the prompt identification of individuals at heightened risk and offers actionable targets for personalized interventions. In terms of future implementation, this model can be integrated into prenatal care as a rapid scoring table during early pregnancy consultations or incorporated into mobile health applications. This approach fosters precise prevention strategies for GDM in maternal health by emphasizing nutrition and health literacy, supplemented by coordinated adjustments in diet, physical activity, and sleep.

## 1. Introduction

Gestational diabetes mellitus (GDM) is characterized as glucose intolerance first identified during pregnancy in women without prior diabetes [1]. Over the last thirty years, its global prevalence has approximately doubled, from 7% to 14%, making it one of the leading metabolic disorders during pregnancy [2]. In China, the average prevalence has been reported at 14.8% (95% CI: 12.8–16.7%), with marked regional variation influenced by genetic predisposition and lifestyle factors [3]. Evidence consistently links GDM with adverse maternal and neonatal outcomes, including preeclampsia, gestational hypertension, macrosomia, and preterm birth [4,5]. In addition, longitudinal studies suggest that both affected mothers and their children face elevated long-term risks of metabolic diseases, supporting the concept of a transgenerational metabolic memory [6,7]. Notably, with the global trend of delayed childbearing age and the escalating obesity epidemic among women of reproductive age [8], the disease burden of GDM continues to rise significantly. Consequently, developing effective risk prediction tools to enable early detection and intervention represents an urgent clinical priority.

Recent studies have been exploring early prediction models for GDM, yet the existing models are mostly limited to demographic indicators and blood biochemical markers [9,10]. Notably, the occurrence of GDM is also influenced by multiple modifiable factors. Accumulating evidence suggests that maternal dietary imbalance, lack of physical activity, poor sleep, and inadequate nutrition and health literacy are strongly related to the onset of GDM. For example, our prior study of 1489 participants in the Tongji Maternal and Child Health Cohort found that stronger adherence to the Chinese Dietary Guidelines for pregnant women was linked to a lower risk of GDM [11]. Consistently, a Korea cohort study (n = 3457) demonstrated that regular physical activity, particularly muscle-strengthening exercises before pregnancy, was protective against GDM [12]. Likewise, findings from a large Chinese cohort (n = 6993) showed that sleeping fewer than 7 h per night during early pregnancy had a significantly greater risk of GDM [13]. In addition to these lifestyle behaviors, nutrition and health literacy serves as the foundation for these behaviors and act as an independent determinant of adverse pregnancy outcomes, including anemia and GDM [14,15]. Therefore, further incorporating modifiable factors in an early prediction model for GDM is of greater practical significance for screening and implementing specific interventions in early and mid-pregnancy.

Current evidence demonstrates that nomogram-based risk prediction models exhibit significant advantages in assessing risks of complex diseases including diabetes, cardiovascular diseases, and cancer [16,17,18]. By integrating multiple factors and their interactions, the nomogram improves predictive accuracy beyond single-factor approaches, thereby facilitating early risk stratification and personalized intervention [16,19]. In addition, the model employs an intuitive graphical interface to transform complex computations into visual outputs, enabling clinicians to observe variable impacts and conduct risk assessments without statistical expertise, thereby enhancing clinical utility [16,17,18,19]. Recent studies in China have demonstrated the utility of nomogram prediction models incorporating GDM risk factors. However, these models predominantly focus on non-modifiable factors (e.g., maternal age, pre-pregnancy body mass index (BMI), family history of disease) [20,21], creating a critical gap as they overlook modifiable factors like dietary quality, physical activity, sleep patterns, and nutrition and health literacy. This limitation restricts their clinical utility for comprehensive prenatal management, especially for pregnant women with inherent non-modifiable risk factors.

Therefore, based on pregnant women at 14–23^+6^ weeks of gestation in the Southern regions of China, this study aimed to construct and validate a nomogram-based risk prediction model for GDM. This model integrates non-modifiable factors (e.g., maternal age, pre-pregnancy BMI, education level, medical history, and family medical history) and modifiable factors (e.g., dietary quality, physical activity, sleep quality, and nutrition and health literacy). We hypothesize that by innovatively integrating modifiable factors, an accurate and clinically applicable model can be established. Compared with previous models, this model can assess GDM risk earlier and more accurately, and intervene in a more targeted and timely manner.

## 2. Methods

### 2.1. Participants

Participants were recruited through a stratified multi-stage random sampling design. Guangdong Province and the Guangxi Zhuang Autonomous Region were selected to represent Southern China. Within each, regions were stratified into urban and rural areas, and two maternal and child health institutions were randomly chosen from each stratum as study sites. As the Maternal and Child Health Care Hospital of Guangxi Zhuang Autonomous Region operates two geographically separate branches, these were treated as independent study units. In total, seven institutions were included.

Recruitment took place from October 2021 to November 2023. Eligible participants were women aged 18–49 years with singleton pregnancies between 14 and 23^+6^ weeks of gestation. Women with pre-pregnancy diabetes, communication barriers, or diagnosed psychiatric disorders were excluded. The study was approved by the Medical Ethics Committee of Tongji Medical College, Huazhong University of Science and Technology (Approval No. 2021-S092) and registered with the Chinese Clinical Trials Registry (ChiCTR2100051019). Written informed consent was obtained from all participants prior to enrollment.

The sample size for this clinical prediction model with a binary outcome (non-GDM vs. GDM) was determined using the events per variable criterion. Based on this method, the effective sample size should include at least 10 events per predictor variable. Considering clinical feasibility, we planned to develop a model with 5 to 10 predictors, requiring a minimum of 100 GDM cases. With a reported GDM prevalence of approximately 14.8% in China [3], the total study population was estimated to exceed 676 participants to ensure adequate statistical power.

### 2.2. Data Collection Process

Information on sociodemographic factors (maternal age, ethnicity, education, household income, and lifestyle/behavior in the first trimester) and clinical factors (maternal and family medical history, conception method, and parity) was retrieved from baseline questionnaires in combination with electronic medical records. Information on pre-pregnancy weight (PPW) was obtained through self-report from participants, whereas anthropometric measurements including body weight and height in the first trimester were obtained by medical staff using calibrated instruments following standardized protocols. According to the filling criteria of the questionnaires, data on dietary quality, physical activity, sleep quality and nutritional health literacy were obtained through face-to-face interviews with trained investigators or self-filling by participants under the guidance of investigators.

#### 2.2.1. The Evaluation Criteria for Pre-Pregnancy BMI

The self-reported PPW of the participants was further calibrated via the PPW prediction model formulated by Thomas, which was employed in our previous study involving a Chinese population [22]. Variables incorporated into the model included first-trimester body weight, gestational age at initial weighing, height, maternal age at conception, and parity. If the variation between reported and estimated weight exceeded ±2 kg, the estimated measurement replaced the self-reported value. Pre-pregnancy BMI was calculated from PPW and height, and classified into four groups: underweight (<18.5 kg/m^2^), normal (18.5–23.9 kg/m^2^), overweight (24.0–27.9 kg/m^2^), and obese (≥28.0 kg/m^2^), in accordance with the Criteria of Weight for Adults (WS/T428-2013) [23].

#### 2.2.2. Dietary Quality Assessment

To capture habitual dietary patterns, the average food intake in the preceding month was measured using a semi-quantitative food frequency questionnaire that has been validated for pregnant populations [24]. To enhance portion-size estimation, standardized tableware and a photographic food atlas developed by our team were used [25]. Daily energy intake was calculated based on the China Food Composition Tables (6th edition) [26]. Overall dietary quality was evaluated with the Chinese Dietary Guidelines Compliance Index for Pregnant Women (CDGCI-PW), a 100-point index developed by our group that includes 13 components reflecting food variety, intake frequency, and nutrient adequacy [11]. Higher scores indicated better adherence to the national dietary guidelines. In this study, dietary quality was classified as “qualified” when both of the following criteria were met: (1) mean daily energy intake between 1755–2145 kcal/day (90–110% of the second-trimester energy requirement), and (2) CDGCI-PW score ≥ 76 [11]. Otherwise, dietary quality was considered “unqualified.”

#### 2.2.3. Physical Activity Assessment

The level of physical activity was assessed through the validated Chinese version of the International Physical Activity Questionnaire–Short Form (IPAQ-S) [27]. This questionnaire captured both the intensity and duration of various activities, including walking, moderate-intensity exercise, vigorous-intensity exercise, and sedentary behavior over the past week. Reported activities were standardized by assigning metabolic equivalent (MET) values, and the cumulative MET score was used to represent total activity. Based on the cutoffs recommended by the IPAQ Working Group, participants were subsequently classified into low, moderate, and high physical activity categories.

#### 2.2.4. Sleep Quality Assessment

The quality of sleep during the preceding month was evaluated using the validated Chinese version of the Pittsburgh Sleep Quality Index (PSQI), which exhibited an internal consistency Cronbach’s α coefficient of 0.845 [28]. This questionnaire includes 18 items that assess multiple dimensions of sleep, producing a total score between 0 and 21. In this scoring system, elevated values correspond to poorer sleep quality. Following conventional cutoff points, participants were divided into Good (≤5), General (6–10), or Poor (≥11) sleep categories.

#### 2.2.5. Nutrition and Health Literacy Assessment

A specialized questionnaire for Chinese pregnant women was developed by our research group to evaluate their nutrition and health literacy. This questionnaire demonstrated good internal consistency (Cronbach’s α = 0.719) [29]. It was organized into three domains: general knowledge and concepts, lifestyle practices, and core skills. It encompassed a total of 24 items, with an overall score ranging from 0 to 96. A score of ≥60% of the maximum possible score was considered qualified, whereas a score below this threshold was considered unqualified.

### 2.3. The Diagnostic Criteria for GDM

GDM was diagnosed by a 75 g oral glucose tolerance test (OGTT) performed at 24–28 weeks of gestation, with diagnostic cutoffs of fasting glucose ≥ 5.1 mmol/L, 1-h glucose ≥ 10.0 mmol/L, or 2-h glucose ≥ 8.5 mmol/L [30].

**Figure 1:**
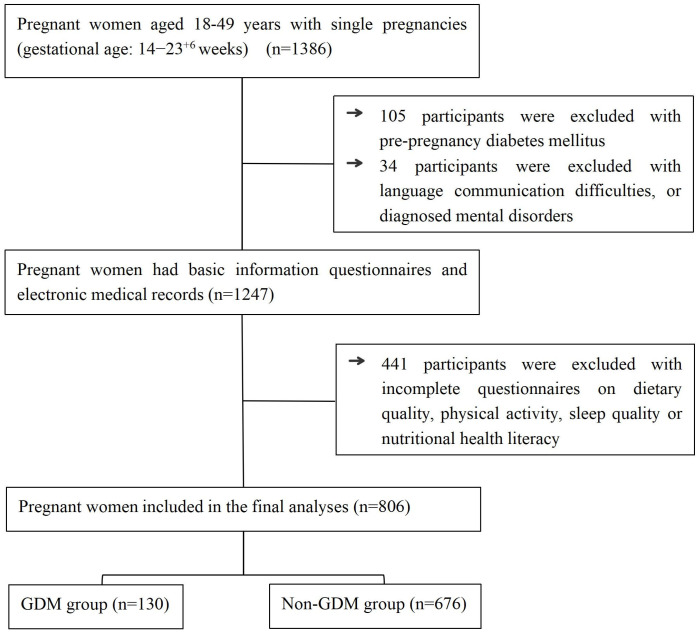
The flowchart of participants’ inclusion.

The flowchart of participants’ inclusion was shown in Figure 1. Eventually, 130 GDM participants and 636 non-GDM participants were included, meeting the requirements for sample size.

### 2.4. Statistical Analysis

All analyses were performed in R software (version 4.2.2, R Development Core Team, Auckland, New Zealand), considering *p* values < 0.05 (two-tailed) as statistically significant. Categorical variables were summarized as frequencies and percentages. Ordered categorical variables were analyzed with the Mann–Whitney U test, while nominal categorical variables were assessed using the Chi-square test. Independent predictors of GDM were identified through multivariable logistic regression, and collinearity among covariates was examined. Participants were randomly assigned to the modeling and validation sets at a ratio of 7:3. A nomogram was constructed using the “rms” package in R. Model performance was evaluated in terms of discrimination, calibration, and clinical utility. Discrimination was assessed with receiver operating characteristic (ROC) curves and the area under the curve (AUC), calibration with calibration plots, and clinical utility with decision curve analysis.

## 3. Results

### 3.1. Comparison of Non-Modifiable Factors

In the final analysis, of the 806 eligible participants, 130 (16.1%) were diagnosed with GDM and 676 (83.9%) were classified as non-GDM. Regarding sociodemographic characteristics, women in the GDM group were more likely to be aged ≥35 years (40.0% vs. 14.9%) and to have been overweight or obese before pregnancy (46.2% vs. 10.5%) compared with those in the non-GDM group, with both differences reaching statistical significance (*p* < 0.001) (Table 1).

Analysis of clinical factors revealed that hypertension (2.3% vs. 0.4%) and polycystic ovary syndrome (PCOS) (19.2% vs. 3.4%) were more common in the GDM group than in the non-GDM group (Table 2). For the other sociodemographic and clinical variables, no significant differences were detected between the two groups (*p* > 0.10) (Table 1 and Table 2).

### 3.2. Comparison of Modifiable Factors

The modifiable factors during 14 to 23^+6^ weeks of gestation included dietary quality, physical activity, sleep quality, and nutrition health literacy. Table 3 shows that, relative to the non-GDM group, pregnant women with GDM were more likely to exhibit adverse modifiable risk factors, including poorer dietary quality (82.3% vs. 72.2%, *p* = 0.016), lower levels of physical activity (46.9% vs. 21.7%, *p* = 0.001), poorer sleep quality (14.6% vs. 3.5%, *p* < 0.001), and lower nutrition and health literacy (90.0% vs. 72.3%, *p* < 0.001).

### 3.3. Multivariate Analysis of GDM

As shown in Table 4, multivariate logistic regression revealed that age, pre-pregnancy BMI, maternal PCOS, dietary quality, physical activity level, sleep quality, and nutrition and health literacy were independent predictors of GDM, with all associations reaching statistical significance (*p* < 0.05). A multiple collinearity diagnosis was performed on all selected predictive factors. The tolerance values of these variables were all >0.90, and the variance inflation factor (VIF) for each was <2.0, indicating a very low degree of collinearity.

### 3.4. Establishment of the Nomogram Model for GDM Risk

**Figure 2:**
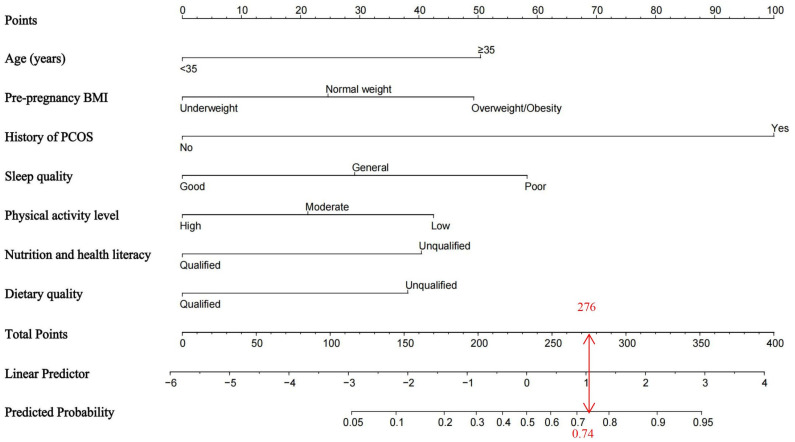
The nomogram prediction model for GDM.

Based on the selected predictors, we established a nomogram model to estimate the risk of GDM (Figure 2). According to this model, the corresponding score for each predictive factor can be derived, and the sum of the scores for all predictive factors corresponds to the probability of a pregnant woman developing GDM. A history of PCOS has the greatest impact on GDM among all selected predictive factors, and modifiable factors (dietary quality, physical activity, sleep quality, and nutrition health literacy) during 14 to 23^+6^ weeks of gestation also play an important role in GDM. For example, if a pregnant woman is under 35 years old, has normal PPW, history of PCOS, general sleep quality, low physical activity level, unqualified nutrition and health literacy, and unqualified dietary quality, her total score can be calculated from the corresponding chart as approximately: 0 + 25 + 100 + 29 + 43 + 41 + 38 = 276. A total score at this level indicated a predicted risk of 0.74 for developing GDM.

A detailed list of the specific values assigned to the predictive factors is shown in Table 5. The model equation is presented as follows: *p* = 1/(1 + exp(−(−0.69 + 1.25 × age + 0.61 × pre-pregnancy BMI + 2.49 × maternal PCOS − 0.73 × sleep quality − 0.53 × physical activity level − 1.01 × nutrition and health literacy − 0.95 × dietary quality)).

### 3.5. Validation of the Nomogram Model for GDM Risk

**Figure 3:**
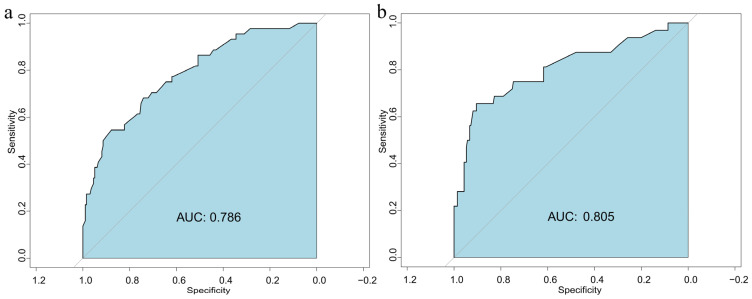
Receiver operator characteristic curves of the prediction model in the modeling (**a**) and validation (**b**) populations.

As illustrated in Figure 3, the AUC values were 0.786 for the modeling group and 0.805 for the validation group, showing only a slight difference and suggesting stable model performance. Since all AUCs exceeded 0.7, the model demonstrated favorable predictive performance.

**Figure 4:**
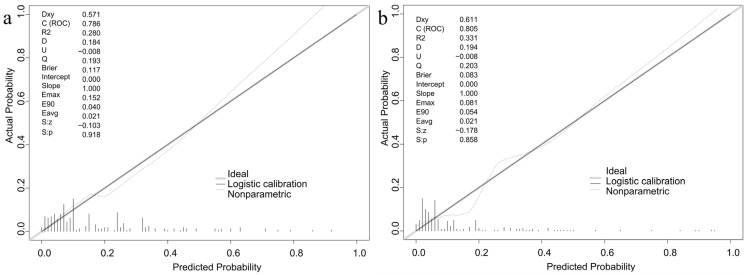
Calibration curves of the prediction model in the modeling (**a**) and validation (**b**) populations.

The calibration curve is presented in Figure 4, with the horizontal axis indicating the predicted risk of GDM and the vertical axis indicating the observed risk. The solid line indicated the ideal predictive capability for GDM risk, while the dashed line represented the actual predictive capability of the model derived from this study. The two lines in the graphs for both the modeling and validation groups were relatively close, demonstrating that the predictive capability of this study was near the ideal level and the calibration degree was within an acceptable range.

**Figure 5:**
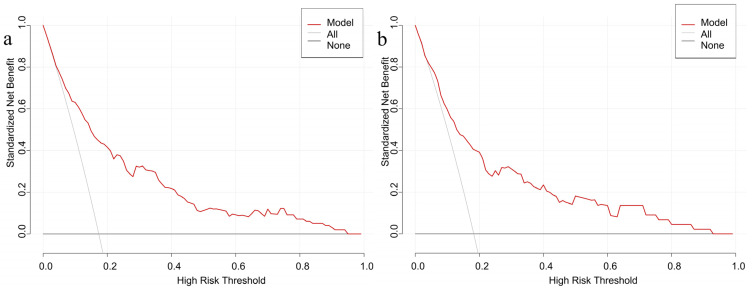
Decision curves of the prediction model in the modeling (**a**) and validation (**b**) populations.

Figure 5 presents the results of the decision curve analysis. For the modeling group, threshold probabilities ranging from 5% to 95%, and for the validation group, from 4% to 92%, showed that applying the nomogram to predict GDM risk provided greater net benefit. These results suggest that the proposed prediction model demonstrates favorable clinical utility.

## 4. Discussion

We constructed and validated a nomogram-based model for predicting GDM that incorporated both fixed risk factors (maternal age, PPW, and PCOS) and modifiable factors (dietary quality, physical activity, sleep quality, and nutrition and health literacy). The model showed consistent predictive capacity across both the training and validation sets, underscoring its applicability in the early detection of GDM within clinical management. Significantly, integrating modifiable variables makes it possible to identify high-risk women and provide precise, preventive interventions.

In our study, the prevalence of GDM was 16.1%, closely matching the pooled national estimate derived from meta-analyses in China [3]. Our predictive model identified 3 non-modifiable risk factors: advanced maternal age, pre-pregnancy overweight/obesity, and maternal PCOS. The deterioration of pancreatic β cell function and exacerbation of insulin resistance with advancing age explain the elevated GDM risk in older women [31]. Our results confirm this, showing a markedly higher prevalence in those aged 35 years or older (40.0% vs. 14.9%, *p* < 0.001), which was consistent with the previous results of Chinese pregnant women [32,33]. This study confirmed that pre-pregnancy overweight/obesity was a highly correlated predictor of GDM, primarily through visceral fat-induced chronic inflammation and insulin resistance [34,35]. While consistent with a cohort study involving 3263 participants, our model assigned a higher coefficient to pre-pregnancy BMI (0.61 vs. 0.061) [33], likely due to changes in the impact of different selected predictive factors. Previous studies have shown that approximately 80% of women with PCOS exhibit intrinsic insulin resistance, and pregnant women with PCOS face a 2- to 4-fold higher risk of developing GDM than those without PCOS [36,37]. However, other evidence suggests that this increased risk may be largely attributable to obesity and advanced maternal age rather than PCOS itself [38]. Earlier risk prediction models also recognized PCOS as having a significant impact on the development of GDM [32]. Consistent with these findings, our study confirmed that a history of PCOS represents a major risk factor for GDM and carried the greatest weighting in our predictive model.

There is substantial evidence that lifestyle modification plays a pivotal role in lowering the risk of GDM [39]. Guided by this understanding, our study examined modifiable determinants such as diet quality, physical activity, and sleep. Poor dietary patterns marked by high consumption of refined carbohydrates and saturated fats together with low fiber intake can exacerbate insulin resistance and impair glucose regulation, thereby increasing GDM susceptibility [40]. A U.S. cohort demonstrated that each one-point increase in the Alternative Healthy Eating Index was associated with an approximately 1% reduction in GDM risk [41]. In our analysis, nutritional status was evaluated through energy intake combined with the CDGCI-PW. We observed that women with higher dietary quality had a lower probability of GDM, and this factor contributed substantially (−0.95) to our predictive model. Moreover, approximately 80% of pregnant women are reported to engage in inadequate physical activity during pregnancy [42], underscoring the urgent need for promoting healthier behaviors in this population. Our study found that lower level of physical activity correlated with higher GDM risk. Chen H et al. similarly reported a significant association between reduced walking time/METs and GDM using the same questionnaire [43]. Xie, Y. et al. further demonstrated that resistance exercise was more effective than aerobic exercise in controlling blood glucose under comparable individualized dietary interventions [44]. These results highlight the need for tailoring intervention strategies for women identified as high risk by our model, particularly in relation to the type of physical activity prescribed. Beyond physical activity, hormonal changes, physiological discomfort, and psychological stress frequently contribute to impaired sleep quality and shortened duration during pregnancy [45]. We observed that women reporting poor sleep quality (PSQI ≥ 11) were more likely to develop GDM, with an incidence of 14.6% compared with 3.5% in those with better sleep. These results are in agreement with a meta-analysis that identified a U-shaped relationship between sleep duration and GDM: both short (<6 h, OR = 1.9, 95% CI: 1.4–2.6) and long (>9 h, OR = 1.5, 95% CI: 1.1–2.0) sleep durations increased risk [46]. The biological mechanisms underlying these associations are biologically plausible. Sleep disturbances promote insulin resistance through hypothalamic–pituitary–adrenal axis activation and cortisol release. In parallel, suboptimal sleep can increase circulating inflammatory mediators (e.g., *C*-reactive protein, IL-6, TNF-α). These cytokines contribute to systemic inflammation, reduce insulin sensitivity, and disrupt insulin signaling, ultimately leading to impaired glucose metabolism [47,48]. Taken together, these results highlight sleep as an often neglected but clinically important lifestyle factor influencing maternal metabolic health. Incorporating sleep assessment and intervention, such as improving sleep hygiene and screening for sleep disorders, into prenatal care should be considered as part of a broader strategy for GDM prevention.

In pregnancy, nutrition and health literacy encompasses the ability of women to acquire, comprehend, and apply essential information related to diet and health. Adequate literacy not only influences maternal and neonatal health outcomes but also contributes to long-term developmental potential and societal advancement [49]. The uneven economic development across regions and the lack of standardized questionnaires may contribute to regional differences in maternal health literacy levels, yet overall, Chinese pregnant women exhibit relatively low health literacy level [50,51]. In this study, the overall nutrition and health literacy qualification rate of the population was only 24.8%, with merely 10% in the GDM group. A synergistic relationship exists between nutrition and health literacy and key lifestyle factors, including diet, physical activity, and sleep. Greater nutrition and health literacy fosters better comprehension of nutritional guidance and promotes healthier, nutrient-dense food choices [52,53]. It also supports positive lifestyle behaviors, including regular physical activity, improved sleep quality, and greater self-efficacy [54,55]. Therefore, we suggest that for GDM high-risk pregnant women screened using this model, improving their nutrition and health literacy levels should be the core, combined with coordinated interventions of diet, exercise, and sleep.

Several limitations of this study should be noted. Firstly, the sample was only from the southern region of China, which may limit the model’s generalizability. Secondly, using questionnaires to collect data on diet, physical activity, sleep, nutrition and health literacy may lead to recall bias, and evaluating these modifiable factors at a single time point may overlook their inherent changes during pregnancy. Thirdly, potential confounding from unmeasured psychological stress and environmental factors could not be ruled out. Finally, some objective biomarker indicators such as inflammatory factors were not included. Future research should conduct multi-regional external validation, portable monitoring devices for physical activity and sleep to obtain objective indicators, and add biomarker indicators to further improve the model. After screening the GDM high-risk population through the model, how to effectively carry out comprehensive interventions targeting modifiable factors is also the direction of future research.

## 5. Conclusions

In summary, this study constructs a GDM risk prediction model that integrates multidimensional factors. Compared with previous studies, this study specifically considers modifiable factors, which provide important insights for early prevention and control of GDM. It also lays an important foundation for developing personalized risk management tools, achieving dynamic monitoring and precise intervention of risks, and establishing a more effective GDM prevention and control system. In terms of future clinical implementation, this model can be integrated into prenatal care as a rapid scoring table during early pregnancy consultations or incorporated into mobile health applications to facilitate real-time risk assessment and timely intervention.

## Figures and Tables

**Table 1:** Distribution of sociodemographic factors among pregnant women.

Characteristics	Non-GDM (n = 676)		GDM (n = 130)		*p*
	n	%	n	%	
**Age (years)**					<0.001
<35	575	85.1	78	60.0	
≥35	101	14.9	52	40.0	
**Pre-pregnancy BMI (kg/m^2^)**					<0.001
Underweight	109	16.1	23	17.7	
Normal weight	496	73.4	47	36.2	
Overweight/Obesity	71	10.5	60	46.2	
**Ethnicity**					0.270
Han	160	23.7	25	19.2	
Minority	516	76.3	105	80.8	
**Education level**					0.728
Junior high school and below	203	30.0	44	33.8	
Senior high school	120	17.8	22	16.9	
Junior college/vocational university	156	23.1	25	19.2	
Bachelor’s degree or above	197	29.1	39	30.0	
**Per capita** **monthly income (RMB)**					0.358
<2999	242	30.0	34	26.2	
3000~4999	190	23.6	27	20.8	
5000~9999	240	29.8	42	32.3	
≥10,000	134	16.6	27	20.8	
**Lifestyle and behavior in the first trimester**					
Exercise	245	36.2	48	36.9	0.883
Insomnia	105	15.5	19	14.6	0.119
Smoking	3	0.4	1	0.8	0.629
Drinking	43	6.4	12	9.2	0.235
**Mode of conception**					0.767
Spontaneous	755	93.7	121	93.1	
Assisted reproductive technology	51	6.3	9	6.9	
**Parity**					0.723
Primiparous	427	53.0	72	55.4	
Secundiparous	279	34.6	41	31.5	
Multiparous (parity ≥ 3)	100	12.4	17	13.1	

**Table 2:** Maternal and familial medical history of pregnant women.

Variables	Non-GDM (n = 676)		GDM (n = 130)		*p*
	n	%	n	%	
**Maternal medical history**					
Hypertension	3	0.4	3	2.3	0.024
Thyroid disease	61	9.1	10	7.7	0.491
Polycystic ovary syndrome	23	3.4	25	19.2	<0.001
**Familial medical history**					
Obesity	48	7.1	11	8.5	0.585
Diabetes mellitus	67	9.9	15	11.5	0.574
Hypertension	124	18.3	23	17.7	0.860
Hyperlipidemia	43	6.4	9	6.9	0.811
Polycystic ovary syndrome	44	6.5	8	6.2	0.880

**Table 3:** Dietary quality, physical activity level, sleep quality and nutritional health literacy of pregnant women.

Variables	Non-GDM (n = 676)		GDM (n = 130)		*p*
	n	%	n	%	
**Dietary quality**					0.016
Qualified	188	27.8	23	17.7	
Unqualified	488	72.2	107	82.3	
**Physical activity level**					0.001
High	200	29.6	22	16.9	
Moderate	329	48.7	47	36.2	
Low	147	21.7	61	46.9	
**Sleep quality**					<0.001
Good	433	64.1	65	50.0	
General	219	32.4	46	35.4	
Poor	24	3.5	19	14.6	
**Nutrition and health literacy**					<0.001
Qualified	187	27.7	13	10.0	
Unqualified	489	72.3	117	90.0	

**Table 4:** Multi-variable logistic regression analysis for independent predictors of GDM.

Variables	β	*p*	OR (95% CI)	VIF	Tolerance
Age	1.138	<0.001	3.119 (1.912–5.075)	1.090	0.917
Pre-pregnancy body mass index	0.760	<0.001	2.138 (1.462–3.149)	1.097	0.912
Maternal hypertension	1.741	0.099	5.702 (0.629–45.693)	1.016	0.984
Maternal Polycystic ovary syndrome	2.205	<0.001	9.074 (4.416–18.831)	1.011	0.989
Dietary quality	−0.639	0.019	0.527 (0.302–0.889)	1.013	0.987
Physical activity level	−0.386	0.012	0.679 (0.499–0.918)	1.013	0.987
Sleep quality	−0.961	<0.001	0.382 (0.266–0.545)	1.019	0.981
Nutrition and health literacy	−1.595	<0.001	0.202 (0.096–0.390)	1.009	0.991

VIF: variance inflation factor.

**Table 5:** A detailed list of the specific values assigned to the predictive factors.

Predictive Factors	Classifications	Values
Age (years)	<35	0
	≥35	1
Pre-pregnancy body mass index (kg/m^2^)	Underweight	1
	Normal weight	2
	Overweight/Obesity	3
Maternal Polycystic ovary syndrome	No	0
	Yes	1
Dietary quality	Qualified	1
	Unqualified	0
Physical activity level	High	3
	Moderate	2
	Low	1
Sleep quality	Good	3
	General	2
	Poor	1
Nutrition and health literacy	Qualified	1
	Unqualified	0

## Data Availability

The data presented in this study are available on request from the corresponding author due to confidentiality (collaboration agreements).
